# Dental Mechanism

**Published:** 1884-05

**Authors:** A. Retter


### DENTAL MECHANISM.

BY A. RETTER, D. D. S.

READ BEFORE THE FIFTH DISTRICT DENTAL SOCIETY OF THE STATE OF NEW YORK, UTICA, APRIL 8 AND 9, 1884.

Rubber is the most abused of all the dental agents employed in mechanical dentistry.

It has been brought into such disrepute by the bungling work of cheap dentists, that many intelligent practitioners have either abandoned its use, or are seriously contemplating such a step. They claim that this material has been the chief cause of the decadence of mechanical dentistry, because of its cheapness and its apparently easy manipulation, which enables any boy or novice to plaster up a set of teeth at a nominal cost, and that in order to restore mechanical dentistry to its former position rubber must be abandoned, and gold, which is more expensive and more difficult to manipulate, must be adopted.

Moreover, it is also claimed that rubber is a non-conductor, and therefore the cause of sore mouths, and of lasting injury to the oral tissues.

While I believe that rubber is greatly abused, I maintain that it is one of the most useful agents we have in mechanical dentistry; that no laboratory is complete without it, and that it cannot and never should be abandoned, unless another material equally as good and as readily manipulated is invented.

The great mass of the people will not and cannot afford the high-priced bases, such as gold and continuous gum, nor can as good average results be obtained by them as readily as with rubber.

Continuous gum, gold, aluminum, Rees's metal, and celluloid, each have their good qualities, but rubber too has its many advantages, with fewer disadvantages than almost any of the other bases.

It is the most plastic material we have, which property enables a direct application to the model, and assures a good fit when properly manipulated. It is as elastic a material as we have, even more so than gold, and it is also the strongest and most durable for a properly constructed plate, is almost indestructible, and will never wear out. But it is cheap and a non-conductor, and the cause of cheap work. These attributes have brought upon it the undeserved condemnation of well-meaning dentists, who cannot see that it is left with the operator to make the material valuable or worthless, just as an artist may take a canvas and change its value by the skill and study expended upon it.

I cannot for a moment comprehend why we should insert a rubber plate chiefly because the material is cheap, or because some charlatan sets his standard of skill even with or lower than the value of the material. What regular physician would not scout the idea of charging less for his visit because the medicine used was cheaper? Does he not charge for his knowledge, for his study of the case, and for knowing what medicine to give to effect a cure? And why should not dentists do the same? Certainly the construction of a first-class denture requires experience, thought and study.

A boy or novice may soon be able to pack and vulcanize a plate, and file and scrape it; but is this dentistry? Is this all there is in the entire construction of an artificial denture, beginning with the impression and ending with the insertion of a well-fitted and well-articulated plate?

To obtain a nice impression in a delicate and neat manner is by no means an easy task, to say nothing of the study of the model, arrangement of the teeth, and their selection; for we must restore the features of the face, and the expression marred by the loss of the teeth. To do all this properly is not within the province of a novice or a boy, but it requires years of experience, besides considerable natural talent and ability.

It is the duty of every dentist to make use of that material which in general will produce the best results, whether it be rubber, celluloid, or gold.

The dentist who will recommend a high-priced material simply because it increases the bill is doing as great a wrong as the five-dollar bungler. Nor will gold or other high-priced materials ever elevate mechanical dentistry. I believe that the only way to do this is to give to it our best thought and careful study, and produce superior results. The trouble with rubber or celluloid is not in the material, but in the dentist; let him take pains to elevate his work, and he will elevate himself and dentistry.

Perhaps there would be but few dentists who would not more willingly acknowledge the excellency of rubber were it not for its non-conducting property, which, it is argued, is the cause of sore mouths.

Rubber only too often receives the blame which should be laid in many instances to other causes. I have met with sore mouths under gold, platina, and continuous gum. Indeed, one of the worst cases I ever saw was caused by a partial gold plate, grinding and wearing against the natural teeth. I much prefer for a partial denture a narrow, well-vulcanized, elastic band of rubber, with well-fitted gold clasps or stays; such a piece is lighter and much pleasanter to wear, and less injurious to adjoining teeth. It is surprising how in these cases a little thought and study over a model will bring out ways and means to avoid a large plate, and the covering up of tissues.

Whenever a sore mouth is produced by a rubber plate, in a large majority of cases it can be traced to other causes, such as a want of cleanliness, poor quality of the rubber, and imperfect vulcanization. Rubber loaded with kaolin, or other earths, and not sufficiently or properly vulcanized, soon becomes rotten and filthy, and a source of irritation. In fact, I have noticed but very few cases of a properly fitted plate of well vulcanized, superior rubber, where inflammation could absolutely and solely be traced to the heat produced through the surface of the mouth being covered by a non-conductor. On very soft and flabby gums nearly all of the different bases will produce inflammation, if not kept strictly clean. A gold plate in these cases may be easier to clean, and therefore be less injurious; but if a rubber plate is vulcanized rather hard, and its surface next to the gum and palate nicely polished, it can be kept equally as clean. In all cases of sore mouths I direct the leaving of the plate out at night, and absolute cleanliness, with the use of Phenol Sodique as a wash.

To obtain a good rubber plate, a good quality of rubber must be used. Of all the different kinds, I have found none as good nor as strong as the Akron rubber. The plaster model should never be subjected to boiling water, whether for cleaning or for bringing the flasks together after packing, both of which can readily be done without its use.

I pack in the new mode heater, using dry heat, and this apparatus I consider so excellent for the purpose that I would have one even if it were useless for any other purpose. Rubber or celluloid will never get into the joints of gum-blocks when packed in the new mode with anything like care. I prefer to use plenty of rubber, with strong pressure. The flasks should never be immersed in water when in the vulcanizer. I place an old, upper half flask, or rim in the bottom of the vulcanizer, and fill with water just to its upper edge, then put in my flasks, heat up slowly, and vulcanize at 320 degrees, one hour and ten minutes. If a set is thus packed and vulcanized without subjecting it to boiling water, the result is a strong, tough, and dense rubber, and no broken blocks.

For celluloid I also prefer the new mode, and for full sets prefer this material in many cases. If care is taken, and a good, well seasoned blank can be obtained, it makes a much more beautiful set than rubber, and probably equally as good. I take two impressions, and make two models, and when I have the teeth arranged and waxed, I wax my second model as I would like to have my blank in shape and size, then invest in a flask, remove the wax and press a suitable blank to shape. When cool, I trim it and use it for the plate. I raise the heat to 320 degrees, and take great pains to get the plate out in such a manner that but a trifling filing and finishing has to be done. To obtain a good celluloid plate, the waxing must be done so neatly that the plate when cut from the plaster will be ready for the cone and brush. In many cases where I have substituted celluloid plates for rubber, the patients have been much gratified with the result, especially when they were wearing poorly made rubber plates. However, I had also several cases where a first-class rubber plate had been worn for years, and when I substituted celluloid the patients assured me that they could absolutely feel no difference, but they liked its appearance better. I prefer plain teeth for all celluloid work.

